# Identification and validation of calcium signaling pathway-related biomarkers in T1 and T2 lymph node metastatic gastric cancer

**DOI:** 10.3389/fgene.2025.1653700

**Published:** 2025-10-01

**Authors:** Mingzhi Cai, Xinyang Nie, Fenglin Cai, Xiuding Yang, Weilin Sun, Rupeng Zhang, Han Liang, Yonglin Yang, Li Zhang

**Affiliations:** ^1^ Department of Gastric Surgery, Tianjin Medical University Cancer Institute and Hospital, National Clinical Research Center for Cancer, Tianjin Key Laboratory of Digestive Cancer, Tianjin’s Clinical Research Center for Cancer, Tianjin, China; ^2^ Department of General Surgery, Gannan Tibetan Autonomous Prefecture People’s Hospital, Lanzhou, Gansu, China

**Keywords:** early gastric cancer, lymph node metastasis, calcium signaling pathway, biomarkers, RET

## Abstract

**Background:**

Lymph node (LN) status is crucial for assessing the treatment effectiveness and potential for cure in early gastric cancer (GC; T1-T2), whether treated through endoscopy or surgery. The purpose of this study was to identify biomarkers related to calcium signaling pathway in T1 and T2 lymph node metastatic gastric cancer and explore potential regulatory mechanisms.

**Methods:**

All data applied in this study were obtained from public databases. Biomarkers were identified through univariate Cox regression analysis and survival analysis. Subsequently, enrichment analysis, somatic mutation analysis, immune microenvironment analysis, drug sensitivity analysis, and single cell analysis were used to investigate the functional mechanisms. Finally, clinical sample validation was performed.

**Results:**

RET was identified as a biomarker through selection. Enrichment analysis indicated that 36 significantly different pathways between the NP (LN-positive samples (N1, N2, N3)) and NO (LN-negative samples (N0)) groups. A total of 2 oncogenic pathways showed significant differences between the NP and NO groups. The scores of 14 immune cell types showed significant differences, including mast cells. RET exhibited the strongest correlation with mast cells. The ESTIMATE score, stromal score, and immune score were significantly elevated in the NP group. Additionally, the NP group showed significantly higher expression of 13 immune checkpoint genes. TP53 had the highest mutation rate in both the NP and NO groups. There was a significant difference in the sensitivity to 15 chemotherapy drugs between the NP and NO groups. Additionally, RET was expressed in multiple cell types, including fibroblasts and mast cells. In both the TCGA-GC-LN and GSE84433 datasets, RET was significantly upregulated in the NP group. The RT-qPCR results of clinical samples also indicated a significant upregulation of RET in the NP group.

**Conclusion:**

RET laid the foundation for targeted therapy in the treatment of T1 and T2 lymph node metastatic gastric cancer.

## 1 Introduction

Gastric cancer ranks among the malignancies with the highest global incidence and mortality rates (Smyth et al., 2020). According to the TNM staging system, the T stage indicates the extent of tumor infiltration into the gastric wall. Specifically, T1 stage denotes that the tumor is confined to the mucosa or submucosal layer, while T2 stage signifies that the tumor has penetrated the submucosal layer but remains restricted to the muscularis propria (Kumagai and Sano, 2021). Lymph node metastasis, represented by the N stage, serves as an independent prognostic factor that substantially influences patient survival outcomes (Dai et al., 2024; Imtiaz et al., 2023; Dong et al., 2023). The rates of lymph node metastasis for T1 and T2 stage gastric cancer are considerably lower than T3/T4 stages (Nie et al., 2025). Nonetheless, metastases in T1 and T2 stages are predominantly localized to regional lymph nodes and are more insidious (Imtiaz et al., 2023). Regarding treatment options, patients with T1N0 gastric cancer may opt for endoscopic submucosal dissection (ESD), whereas those with T1 or T2 tumors accompanied by lymph node metastasis require radical gastrectomy combined with D1/D2 lymph node dissection, followed by adjuvant chemotherapy or targeted therapy. Clinical trials have demonstrated that laparoscopic sentinel lymph node navigation surgery offers comparable prognostic outcomes to conventional D2 radical surgery for cT1-T2N0 gastric cancer (Kim et al., 2022). This approach is advantageous in preserving gastric function and minimizing resection, thereby enhancing patients' quality of life and nutritional status, contingent upon a thorough assessment of lymph node involvement (Kitagawa et al., 2013). Nevertheless, the current diagnostic and therapeutic framework encounters several challenges: excessive lymph node dissection in early-stage patients may increase the risk of complications, while insufficient dissection could result in undetected metastases. Additionally, traditional imaging modalities exhibit limited sensitivity in identifying micrometastases and lack molecular targeting strategies for lymph node metastasis in T1 and T2 stage gastric cancer.

The Calcium Signaling Pathway functions as a pivotal second messenger system within cells, primarily involving calcium ions (Ca^2+^) (Li et al., 2025; Kong et al., 2021). This pathway is integral to maintaining the dynamic equilibrium of calcium ions both intra- and extracellularly, as well as among various organelles. It is crucial for several physiological processes, including muscle contraction, nerve conduction, cell proliferation, and differentiation (Sukumaran et al., 2021). The fundamental mechanism of this pathway entails the coordinated action of calcium ion channels, calcium pumps, and calcium-binding proteins (Robitaille and Monteith, 2025). These components integrate external stimuli via multiple pathways, such as the phosphatidylinositol signaling pathway and the MAPK pathway, to regulate cellular functions (Wu et al., 2021). Recent research has indicated that dysregulation of the calcium signaling pathway is intricately associated with tumorigenesis (Zheng et al., 2023). In gastric cancer, aberrant activation of the calcium signaling pathway is significantly associated with metastatic potential (Wang et al., 2024; Inoue et al., 2022; Wang et al., 2022; Wu et al., 2021). Recently, Wu et al. identified ORAI2—a poorly characterized subunit of store-operated calcium (SOC) channels—as a key regulator of lymph node metastasis in gastric cancer, demonstrating its upregulation promotes tumor cell proliferation and migration via calcium-dependent signaling (Wu et al., 2021). While current research has elucidated certain mechanisms of the calcium signaling pathway in the context of lymph node metastasis in gastric cancer, the precise regulatory network involved in T1/T2 staging remains to be thoroughly investigated.

This study employed transcriptome data of lymph node metastasis in T1 and T2 stage gastric cancer, obtained from public databases, and integrated it with genes related to calcium signaling. Biomarkers were identified through differential expression analysis, univariate Cox regression analysis, and survival analysis to elucidate their predictive value for lymph node metastasis in T1 and T2 stage gastric cancer. Furthermore, the study investigates the biological functions of these biomarkers, their associations with the immune microenvironment, somatic mutations, drug sensitivity, and the molecular regulatory networks involved, as well as their expression within the cellular environment. These findings aim to provide novel insights into the diagnosis and treatment of lymph node metastasis in T1 and T2 stage gastric cancer.

## 2 Methods

### 2.1 Data sources

The TCGA-STAD dataset was downloaded from The Cancer Genome Atlas (TCGA) database (https://portal.gdc.cancer.gov/) (access on 2024) as a transcriptome dataset for gastric cancer (GC). In this dataset, primary tumor samples from GC patients at T1 and T2 stages were extracted, with samples lacking T1/T2 staging information excluded. Based on the lymph node (LN) metastasis status of these samples (NX samples were excluded), the GC patient samples were divided into LN-positive samples (N1, N2, N3) as the NP group and LN-negative samples (N0) as the NO group, consisting of 49 and 40 samples, respectively (Samples with follow-up periods of less than 30 days during clinical evaluation and those lacking survival information were excluded). The resulting dataset was named TCGA-GC-LN. Simultaneously, somatic mutation data, tumor mutation burden (TMB), and clinical data were downloaded. The GSE84433 (GPL6947) dataset was downloaded from the GEO as a validation cohort. A total of 28 tumor samples from GC patients in T1/T2 stages with LN-positive metastasis were selected as the NP group, while 18 tumor samples from GC patients in T1/T2 stages with LN-negative metastasis were selected as the NO group. Additionally, 254 calcium signaling pathway-related genes (CSP-RGs) (Supplementary Table S1) were obtained from KEGG database.

### 2.2 Differential analysis

DEGs between the NP and NO groups in the TCGA-GC-LN dataset were identified using the DESeq2 package (v 1.38.0) (Huang A. et al., 2024), using thresholds of *P* < 0.05 and |log_2_ FC| > 0.5. A volcano plot of these DEGs was generated using the ggplot2 package (v 3.4.4) (Gustavsson et al., 2022), with annotations for the top 10 upregulated and downregulated genes. A heatmap of the top 10 upregulated and downregulated genes was generated using the ggplot2 package (v 3.4.4).

### 2.3 Candidate genes acquisition and enrichment analysis

DEGs and CSP-RGs were integrated using the ggvenn package (v 0.1.9) (Zhou et al., 2025) to identify the candidate genes. Subsequently, functional enrichment analysis of the candidate genes was carried out with the clusterProfiler package (v 4.7.1.003) (Chen and Boutros, 2011), including Gene Ontology (GO) (*P* < 0.05) and KEGG enrichments (*P* < 0.05). The top 5 terms for each category and the top 11 KEGG pathways were displayed based on *P-*value. Additionally, a protein-protein interaction (PPI) network was established based on candidate genes using the STRING database (confidence score ≥0.4).

### 2.4 Biomarkers acquisition

Univariate Cox regression analysis was performed on the candidate genes in the TCGA-GC-LN dataset using the survival package (v 3.5–3) (Ni et al., 2023) to identify genes linked to overall survival (OS) (HR ≠ 1, *P* < 0.2). The results were visualized using a forest plot created with the forestplot package (v 3.1.1). The proportional hazards (PH) assumption test (*P* > 0.05) was then applied to the univariate Cox analysis results, and genes passing the PH assumption were selected as candidate biomarkers. Additionally, in both the TCGA-GC-LN and GSE84433 datasets, the “survv_cutpoint” function from the “survminer” R package was employed to ascertain the optimal cut-off value for each gene expression level using the maximum selection rank statistical method. Subsequently, based on the determined optimal cut-off value for each candidate biomarker expression, the samples were categorized into high expression and low expression groups. Survival analysis, conducted using the survival package (v 3.5–3), compared survival differences between 2 groups (*P* < 0.05), and Kaplan-Meier (KM) survival curves were plotted using the survminer package (v 0.4.9) (Shi et al., 2023). Genes that exhibited significant survival differences between the 2 groups and consistent survival trends were selected as biomarkers.

Besides, In the TCGA-GC-LN dataset (excluding samples with missing clinical information), the differences (*P* < 0.05) in biomarkers expression between different clinical subgroups were compared using the Wilcoxon test.

### 2.5 Investigation of signal pathways

The reference gene set used was the “c2.cp.kegg.v7.4.symbols.gmt” file from MSigDB. Differential analysis of all TCGA-GC-LN dataset samples was carried out with the DESeq2 package (v 1.38.0) to detect differences between the NP and NO groups. The log_2_FC was calculated, and the genes were sorted by log_2_FC from largest to smallest. GSEA was performed using the clusterProfiler package (v 4.7.1.003) to identify enriched pathways (|NES| > 1, FDR <0.25, and *P* < 0.05). The top 5 most significant pathways were visualized based on *P-*value.

The oncogenic pathways were obtained from the literature (Li et al., 2024) (Supplementary Table S2), and the ssGSEA algorithm was used to calculate the oncogenic pathway scores for both the NP group and the NO group across all samples of the TCGA-GC-LN dataset. The differences in scores between the 2 groups were compared using the Wilcoxon test (*P* < 0.05), and the results with significant differences were presented.

### 2.6 Immune microenvironment analysis

The ssGSEA algorithm was used to evaluate the proportions of 28 immune cell types in the TCGA-GC-LN dataset. The Wilcoxon test (*P* < 0.05) was applied to identify differential scores between the NP and NO groups. Spearman correlation analysis was then performed on all TCGA-GC-LN samples to explore the relationships between biomarkers and differential immune cell types, as well as among the differential immune cell types themselves, using the stats package (v 4.2.2) (Aslam et al., 2023) (|cor| > 0.30, *P* < 0.05) (v 2.2.9). The TIDE platform was used to calculate the TIDE score for patients in both the NP and NO groups, with differences compared using the Wilcoxon test (*P* < 0.05). The ESTIMATE package (v 1.0.13) (Bi et al., 2020) was then employed to calculate immune, stromal, and ESTIMATE scores for both groups, and differences between them were assessed.

Immune checkpoint genes were obtained from the literature (Supplementary Table S3) (Liu et al., 2023), and the differences in their expression between the NP group and the NO group were compared using the Wilcoxon test (*P* < 0.05).

### 2.7 Somatic mutation analysis

In the TCGA-GC-LN dataset (Samples lacking somatic mutation information were excluded), somatic mutation data for each patient were obtained, and the Maftools package (v 2.14.0) (Xu et al., 2021) was used to generate a waterfall plot displaying the top 20 most frequently mutated genes. Next, mutation data from the 2 groups were used for TMB analysis, and TMB score was calculated using the maftools package (v 2.14.0). The TMB scores between the 2 groups were compared using the Wilcoxon test (*P* < 0.05).

### 2.8 Drug sensitivity analysis

A total of 138 chemotherapeutic drugs were sourced from the GDSC database, and the pRRophetic package (v 0.5) (Yan et al., 2022) was utilized to calculate the IC50 values for patients in the TCGA-GC-LN dataset. Subsequently, the Wilcoxon test (*P* < 0.05) was employed to explore differences in IC50 values between the 2 groups. The top 3 drugs were displayed, sorted by *P*-value.

### 2.9 Single cell analysis

The expression of biomarkers in different cell types of GC was analyzed using the TISCH database. Additionally, the HR associated with biomarkers mutations in different cancer types were presented.

### 2.10 Regulation network analysis

Transcription factors (TFs) associated with biomarkers were predicted through the mirnet database. The TF-mRNA network was visualized. MicroRNAs (miRNAs) related to biomarkers were predicted using the microcosm database. The StarBase database was then used to predict long non-coding RNAs (lncRNAs) targeting the identified miRNAs. The lncRNA-miRNA-mRNA network was visualized using ggalluvial package (v 3.9.1) (Shi et al., 2023).

### 2.11 Patient specimens

To assess RET mRNA expression levels, gastric cancer samples at T1 and T2 stages, both with (N = 21) and without (N = 21) lymph node metastasis, were collected from patients who underwent curative surgical resection with lymph node dissection in 2024 at Tianjin Medical University Cancer Hospital, Tianjin, China. None of the patients received neoadjuvant therapy prior to gastrectomy. The Institutional Research Ethics Committee of Tianjin Medical University Cancer Institute and Hospital (approval no. bc2022220) sanctioned all experimental procedures involving these samples. Informed consent was obtained for the collection of all samples in accordance with the Declaration of Helsinki.

### 2.12 RT-qPCR

Total RNA was extracted using RNAiso Plus (TaKaRa, Japan), and complementary DNA (cDNA) was synthesized with the TaKaRa Reverse Transcription Kit (TaKaRa, Japan). Quantitative real-time PCR was conducted on the QuantStudio 5 system (Applied Biosystems, Foster City, CA, USA) to quantify mRNA levels, employing TB Green Premix Ex TaqTM II (TaKaRa, Japan). β-actin served as the reference gene for data normalization. The primer sequences were as follows: RET:5′- GTC​CTC​TTG​CTC​CAC​TTC​AAC​G/CCT​GGC​AGT​TTT​CCA​CAC​AGA​C -3’; β-actin: 5′-ATA​GCA​CAG​CCT​GGA​TAG​CAA​CGT​AC/CAC​CTT​CTA​CAA​TGA​GCT​GCG​TGT​G-3′

### 2.13 Cell lines and cell culture

The human gastric cancer cell line AGS was procured from the American Type Culture Collection (Virginia, USA) and cultured in F12K medium supplemented with 10% fetal bovine serum (FBS). The HGC-27 cell line was obtained from the National Infrastructure of Cell Line Resource (Beijing, China) and maintained in RPMI-1640 medium, also supplemented with 10% FBS. Both cell lines were incubated at 37 °C in a humidified atmosphere containing 5% CO_2_. All cell lines utilized in this study underwent short tandem repeat (STR) validation to confirm their authenticity.

### 2.14 shRNA knockdown and transfection

Initially, the cells were seeded into six-well plates. Subsequently, cell transfection was conducted in accordance with the protocol provided by Lipofectamine™ 3000 (Invitrogen, New York, United States). Following an incubation period of 6 h, a complete medium was added to the cells. The empty pSIH1-puro vector served as the corresponding negative control. The shRNA target sequences were as follows: AGG​GTC​GGA​TTC​CAG​TTA​AAT (shRET#1) and ACC​GCT​GGT​GGA​CTG​TAA​TAA (shRET#2).

### 2.15 Colony arrangement assay

The arrangement of colonies was employed to assess the proliferative capacity of the cells. A total of 2,000 cells were seeded into a 6-well plate and incubated at 37 °C with 5% CO_2_. After a period of 2 weeks, the cells were fixed and stained using 4% paraformaldehyde and crystal violet, respectively.

### 2.16 Cell migration and invasion analysis

For the migration assay, a serum-free cell suspension was introduced into the upper chambers of 24-well Transwell plates (8-μm pore size, Corning, NY, USA) at a density of 4 × 10^4 cells per well. The lower chambers were supplemented with culture medium containing 20% FBS. The invasion assay was conducted using chambers pre-coated with Matrigel (BD Biosciences, USA). Following a 24-h incubation period, the inserts were fixed with 4% paraformaldehyde for 30 min and subsequently stained with 0.4% crystal violet for another 30 min. Cell numbers were quantified by averaging counts from three randomly selected fields.

### 2.17 Statistical analysis

R (v 4.2.2) was utilized to conduct the bioinformatics analysis. Differences between the 2 groups were assessed using the Wilcoxon test (*P* < 0.05). Each experiment was independently performed three times. All experimental data were presented as the mean values ±SD. Data analysis was performed using Prism 8.0 software (GraphPad Software), in which *, **and *** indicated *P* < 0.05, *P* < 0.01, *P* < 0.001, respectively.

## 3 Results

### 3.1 Acquisition of 51 candidate genes

**FIGURE 1:**
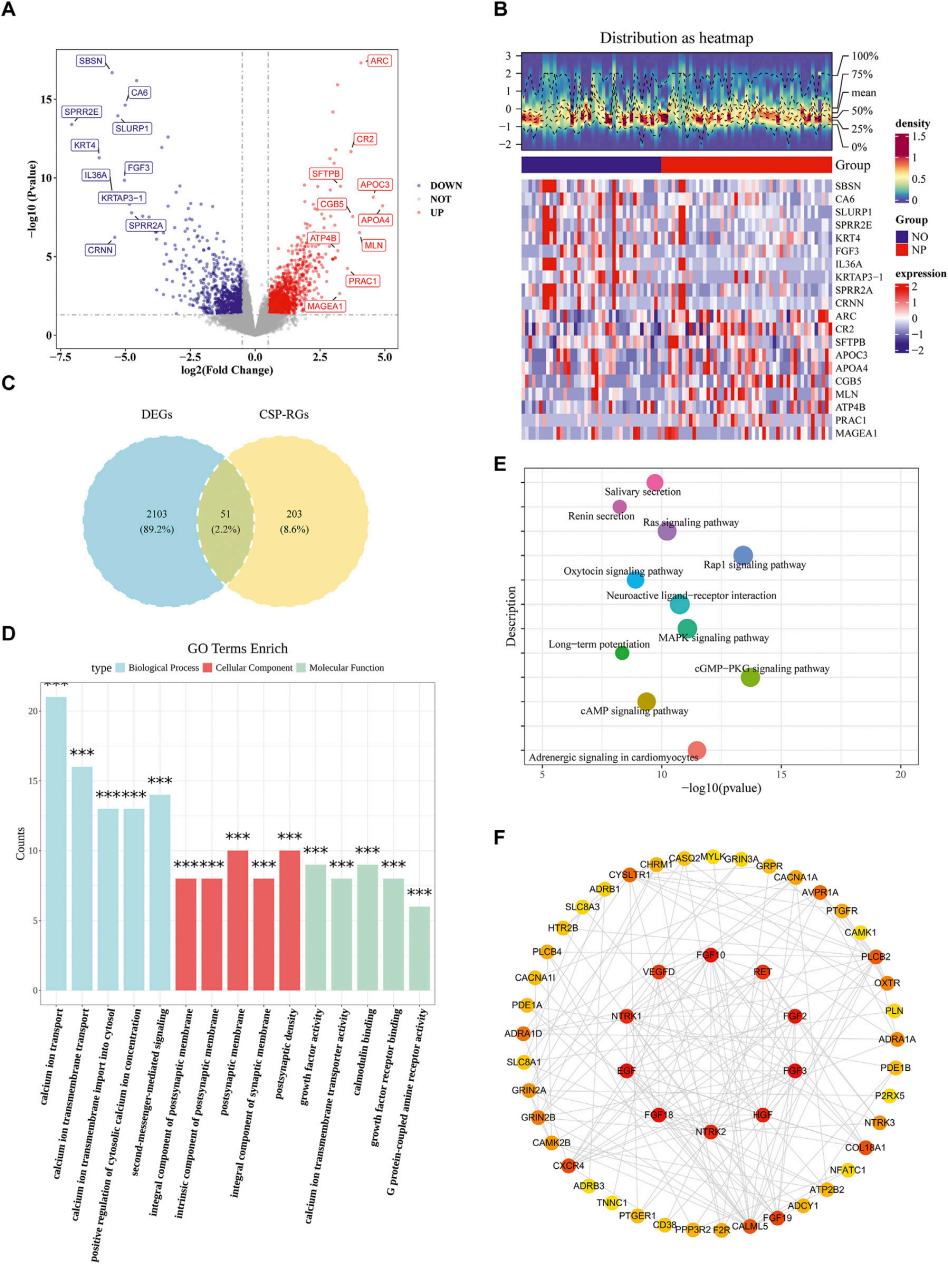
Identification of calcium signaling-related differentially expressed genes (DEGs) in T1/T2 stage gastric cancer. **(A)** Volcano plot of DEGs between lymph node metastasis-positive (NP, n = 49) and -negative (NO, n = 40) gastric cancer samples at T1 and T2 stages from TCGA database (*P* < 0.05, |Log2FC| > 0.5). **(B)** Heatmap of DEGs distinguishing NP and NO groups. **(C)** Venn diagram showing 51 intersecting genes between T1/T2 stage GC-specific DEGs (n = 2,154) and calcium signaling pathway-related genes (CSP-RGs, n = 254), termed Calcium-related Candidate Genes (C-RGs). **(D)** GO enrichment analysis of C-RGs highlighting biological processes. **(E)** KEGG pathway enrichment of C-RGs. **(F)** Protein-protein interaction (PPI) network of C-RGs constructed using STRING (interaction score ≥0.4).

A total of 2,154 DEGs were identified in the TCGA-GC-LN dataset, including 1,334 upregulated genes and 820 downregulated genes in the NP group (Figures 1A,B). By intersecting DEGs and CSP-RGs, 51 candidate genes were selected (Figure 1C). Enrichment analysis revealed that the candidate genes were strongly linked to 1,068 GO terms (*P* < 0.05) and 91 KEGG pathways (*P* < 0.05). For instance, GO terms included “calcium ion transport” and “calmodulin binding” (Figure 1D; Supplementary Table S4). The KEGG pathways primarily included “MAPK signaling pathway” and “cAMP signaling pathway” (Figure 1E; Supplementary Table S5). In the PPI network, a total of 51 proteins were found to interact with each other (such as CALML5 and ADCY1) (Figure 1F).

### 3.2 Identification of RET as biomarkers

**FIGURE 2:**
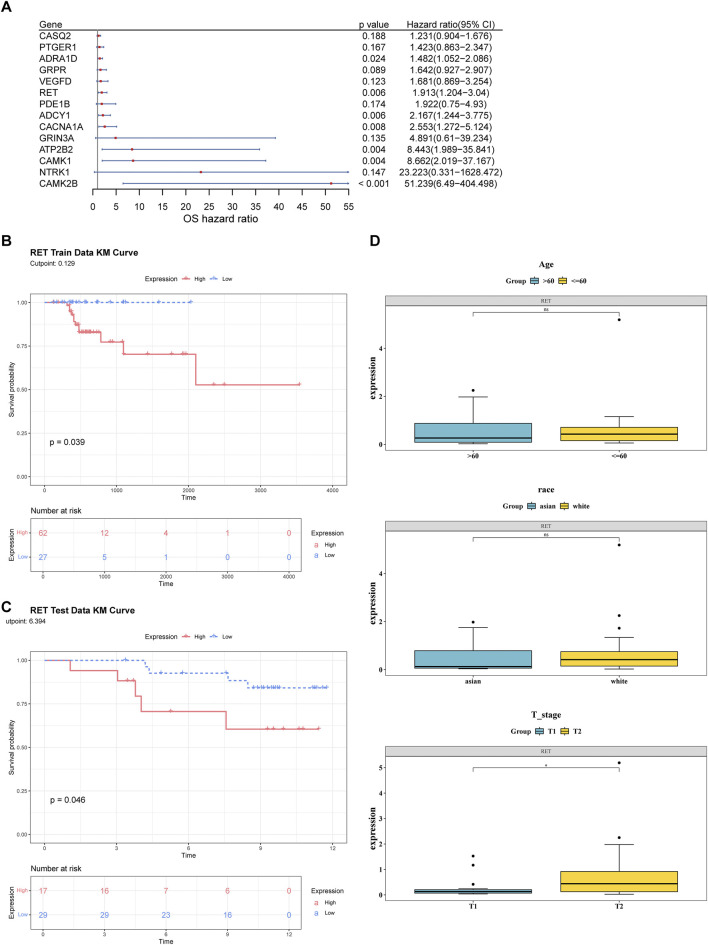
Identification of RET as a prognostic biomarker in T1/T2 gastric cancer. **(A)** Univariate Cox regression analysis of candidate genes in the TCGA-GC-LN cohort (n = 89). **(B,C)** Kaplan-Meier survival curves stratified by high vs low RET expression (log-rank *P* < 0.05). **(D)** Differences in RET expression across clinical subgroups (age, race, T stage).

A total of 14 candidate biomarkers were identified through univariate Cox regression analysis (Figure 2A) and the PH assumption test. In both the TCGA-GC-LN and GSE84433 datasets, the high expression level of RET was associated with poor survival rates and significant differences. Therefore, RET was defined as biomarkers (Figures 2B,C). In addition, RET showed significant differences between the T1 and T2 stages (Figure 2D).

### 3.3 Exploring the signaling pathways of biomarkers

**FIGURE 3:**
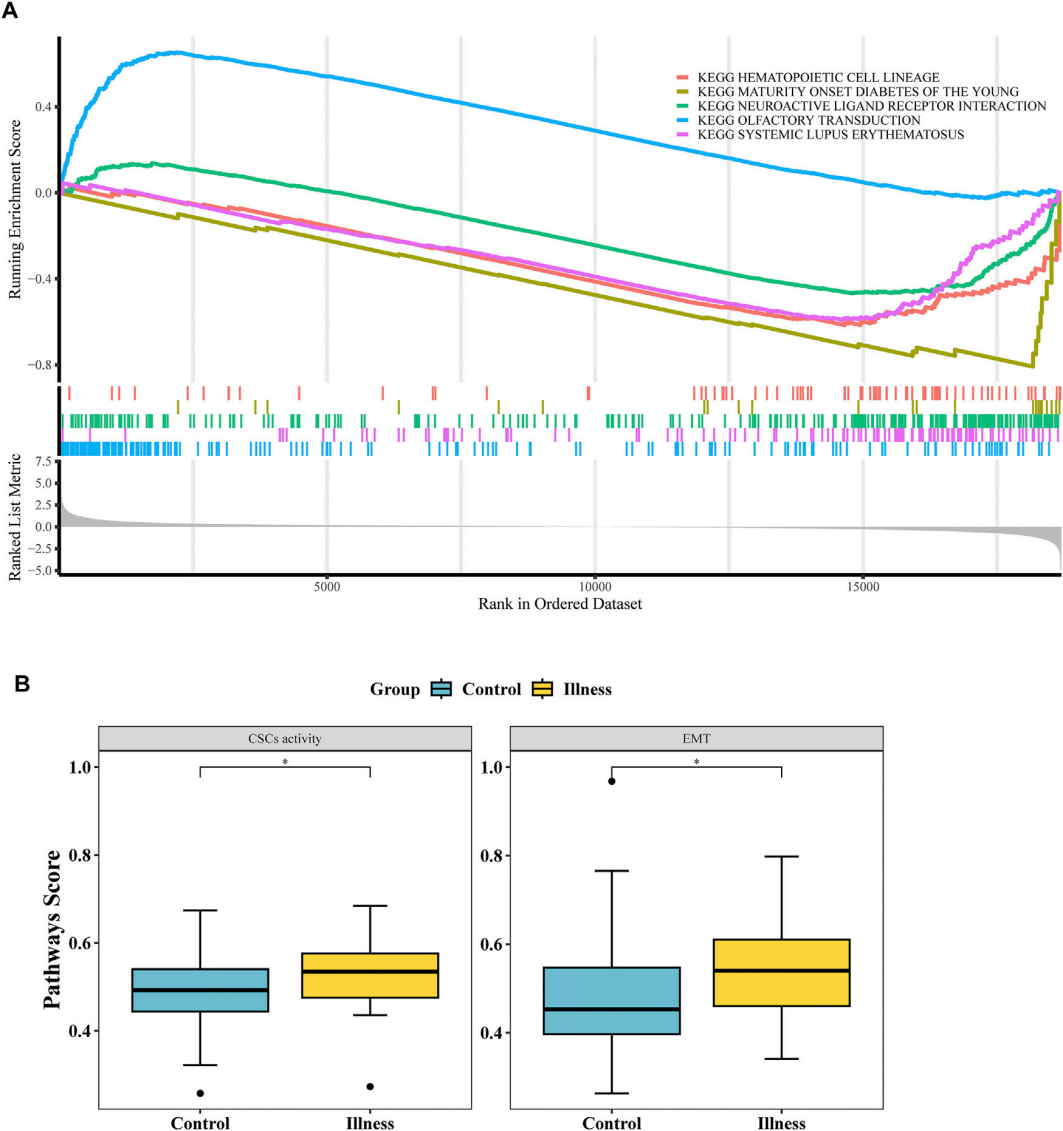
Functional enrichment and signaling pathways associated with LN metastasis. **(A)** Gene Set Enrichment Analysis (GSEA) of 36 hallmark pathways in LN metastasis-positive (NP) vs -negative (NO) groups (|NES| > 1, *P* < 0.05). **(B)** Two oncogenic pathways—Epithelial-Mesenchymal Transition (EMT) and Cancer Stem Cells (CSCs) activity—were significantly upregulated in NP vs NO (*P* < 0.05).

GSEA analysis revealed 36 significantly different pathways between the NP and NO groups (Figure 3A; Supplementary Table S6). Additionally, 2 oncogenic pathways, the “EMT” pathway and the “CSCs activity” pathway, showed significant differences between the NP and NO groups (*P* < 0.05) (Figure 3B). These results suggested that signaling pathways played a crucial role in tumor progression, particularly oncogenic pathways.

### 3.4 Association of biomarkers with the immune microenvironment

**FIGURE 4:**
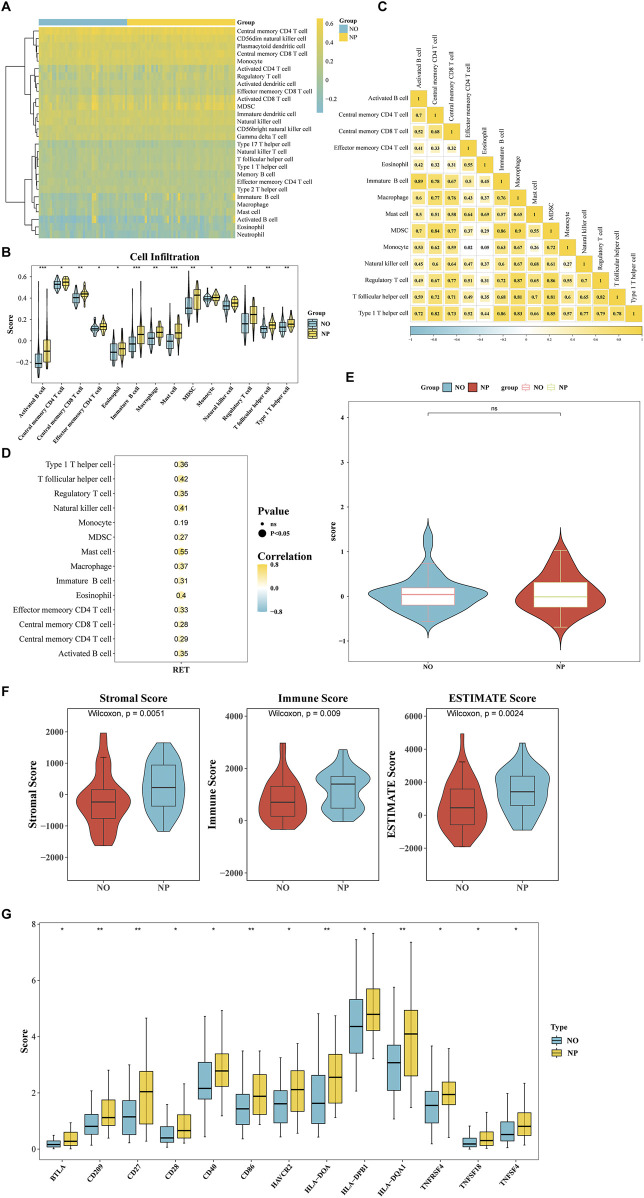
Association of RET with tumor immune microenvironment (TIME) remodeling in T1/T2 gastric cancer. **(A)** The Heatmap demonstrated the percentages of 28 immune cell types in NP and NO groups. **(B)** The scores of 14 immune cell types (*P* < 0.05). **(C)** Correlation heatmap of differentially infiltrated immune cells (|cor| > 0.3, *P* < 0.05). **(D)** RET expression correlation with 14 key immune cells (|cor| > 0.3, *P* < 0.05). **(E)** The tumor immune dysfunction and exclusion (TIDE) scores in NP vs NO (*P* > 0.05). **(F)** Stromal/Immune/ESTIMATE scores significantly higher in NP (all *P* < 0.05). **(G)** Differential expression of 13 immune checkpoint genes (BTLA, CD209, CD27, CD28, CD40, CD86, HAVCR2, HLA-DOA, HLA-DPB1, HLA-DQA1, THFRSF4, TNFSF18, and TNFSF4) (*P* < 0.05).

The Heatmap demonstrated the percentages of 28 immune cell types in NP and NO groups (Figure 4A). The scores of 14 immune cell types showed significant differences, including mast cells (*P* < 0.05) (Figure 4B). The majority of the differential immune cell types exhibited positive correlations, with the strongest correlation observed between monocytes and MDSCs (cor = 0.90, *P* < 0.001) (Figure 4C; Supplementary Table S7). In addition, Spearman correlation analysis revealed that RET exhibited the strongest correlation with mast cells (cor = 0.55, *P* < 0.05) (Figure 4D).

The TIDE score did not show a significant difference between the NP and NO groups (Figure 4E). However, the stromal score (*P* = 0.0051), immune score (*P* = 0.009), and ESTIMATE Score (*P* = 0.0024) were significantly elevated in the NP group (Figure 4F). Additionally, the expression of 13 immune checkpoint genes (BTLA, CD209, CD27, CD28, CD40, CD86, HAVCR2, HLA-DOA, HLA-DPB1, HLA-DQA1, THFRSF4, TNFSF18, and TNFSF4) was significantly higher in the NP group (*P* < 0.05) (Figure 4G). These findings indicated that the tumor microenvironment significantly influenced tumor cells, emphasizing the need to give greater consideration to the tumor microenvironment when developing cancer treatment strategies.

### 3.5 Higher gene mutation rate in the NP group

**FIGURE 5:**
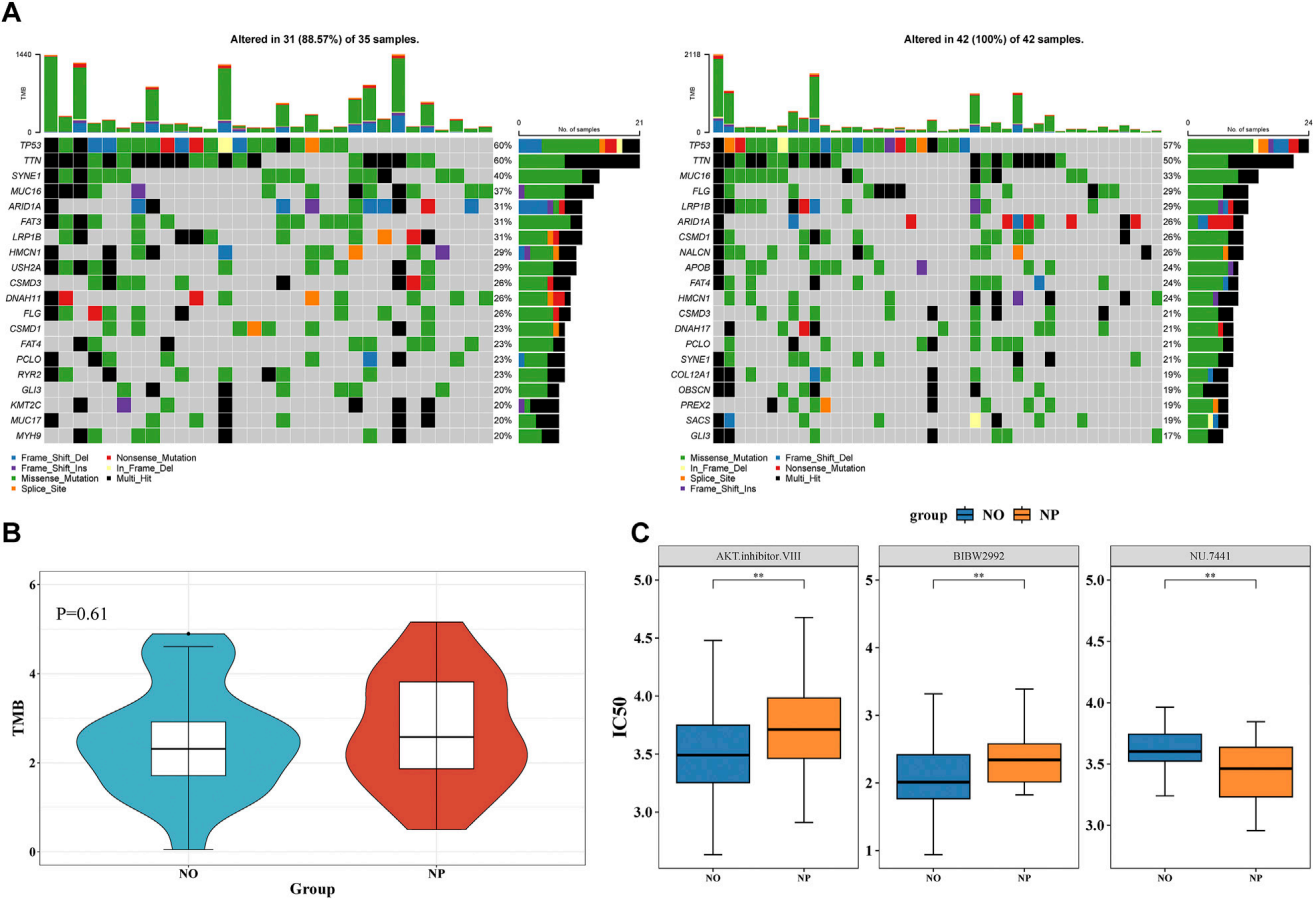
Higher gene mutation rate in the NP group and Investigation of therapeutic drugs **(A)** Waterfall plot of tumor mutational burden (TMB) in NP (n = 49) vs NO (n = 40) groups. **(B)** Wilcoxon test to compare the differences in TMB scores between the NP and NO groups. The TMB score in the NP group was slightly elevated. **(C)** 15 drugs demonstrated significant differences between the NP and NO groups (*P* < 0.05). AKT inhibitor VIII and BIBW2992 had higher IC50 values in the NP group.

TP53 was the gene with the highest mutation rate in both the NP and NO groups, with mutations including missense mutations (Figure 5A). While no significant difference in TMB score was observed, the TMB score in the NP group was slightly elevated, indicating a higher frequency of gene mutations in this group (Figure 5B).

### 3.6 Investigation of therapeutic drugs

In total, 15 drugs demonstrated significant differences between the NP and NO groups. AKT inhibitor VIII and BIBW2992 had higher IC50 values in the NP group, whereas NU.7441 showed a higher IC50 value in the NO group (Figure 5C). These results suggested that these drugs might have played an important role in patients with GC LN metastasis.

### 3.7 Expression of RET in multiple GC cells

**FIGURE 6:**
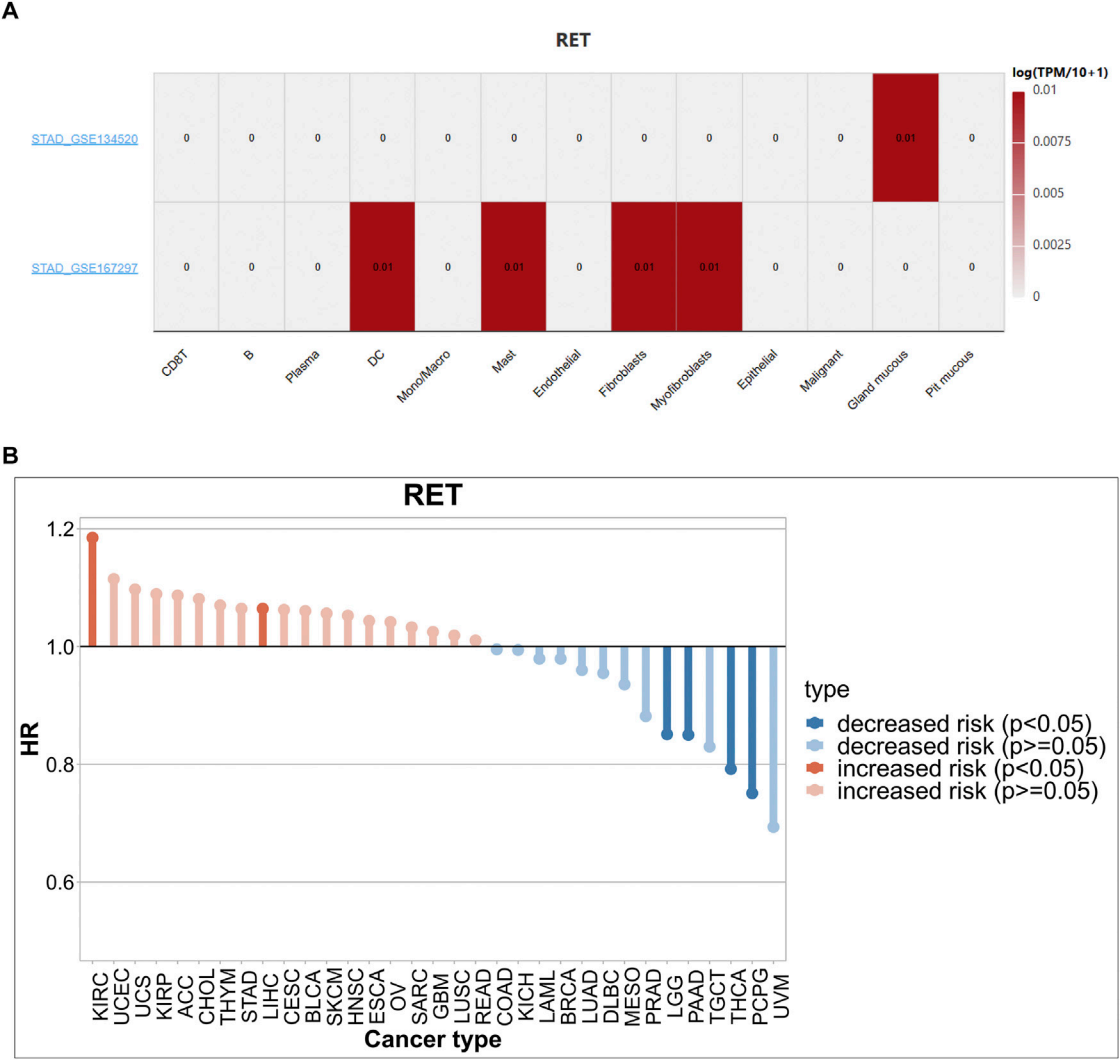
Expression of RET in multiple GC cells **(A)** The expression level of RET in cells in two independent datasets. **(B)** The risk ratio (HR) associated with RET mutations in different cancer types. KIRC, Kidney renal clear cell carcinoma; UCEC, Uterine Corpus Endometrial Carcinoma; UCS, Uterine Carcinosarcoma; KIRP, Kidney renal papillary cell carcinoma; ACC, Adrenocortical carcinoma; CHOL, Cholangiocarcinoma; THYM, Thymoma; STAD, Stomach adenocarcinoma; LIHC, Liver hepatocellular carcinoma; CESC, Cervical squamous cell carcinoma and endocervical adenocarcinoma; BLCA, Bladder Urothelial Carcinoma; SKCM, Skin Cutaneous Melanoma; HNSC, Head and Neck squamous cell carcinoma; ESCA, Esophageal carcinoma; OV, Ovarian serous cystadenocarcinoma; SARC, Sarcoma; GBM, Glioblastoma multiforme; LUSC, Lung squamous cell carcinoma; READ, Rectum adenocarcinoma; COAD, Colon adenocarcinoma; KICH, Kidney Chromophobe; LAML, Acute Myeloid Leukemia; BRCA, Breast invasive carcinoma; LUAD, Lung adenocarcinoma; DLBC, Lymphoid Neoplasm Diffuse Large B-cell Lymphoma; MESO, Mesothelioma; PRAD, Prostate adenocarcinoma; LGG, Brain Lower Grade Glioma; PAAD, Pancreatic adenocarcinoma; TGCT, Testicular Germ Cell Tumors; THCA, Thyroid carcinoma; PCPG, Pheochromocytoma and Paraganglioma; UVM, Uveal Melanoma.

The expression of RET in GC single cells was analyzed using the TISCH database. The results indicated that RET was expressed in multiple cell types, including DC, mast cells, fibroblasts, myofibroblasts, and gland mucous (Figure 6A). In addition, RET mutations increase the risk of cancers such as KIRC, while decreasing the risk of cancers such as UVM (Figure 6B). Fibroblasts were one of the major sources of the extracellular matrix (ECM). During the early stages of gastric cancer development, fibroblasts secreted a large amount of ECM components, such as collagen. Abnormal accumulation and remodeling of the ECM disrupted the normal growth, differentiation, and signaling of gastric epithelial cells, thus creating conditions conducive to the development of early GC.

### 3.8 Exploring the molecular regulatory mechanisms of biomarkers

**FIGURE 7:**
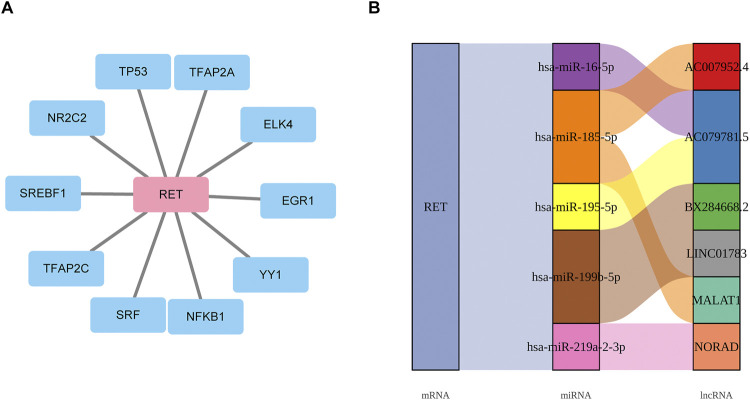
Exploring the molecular regulatory mechanisms of biomarkers **(A)** Transcriptional regulatory network linking 10 TFs to RET mRNA. **(B)** mRNA-miRNA-lncRNA regulatory network.

A total of 10 TFs were found to be associated with RET, including TP53 (Figure 7A). Additionally, 5 miRNAs and 6 lncRNAs were predicted. The identified regulatory networks included MALAT1-hsa-miR-185-5p-RET and AC007952.4--hsa-miR-185-5p-RET (Figure 7B). These potential regulatory mechanisms had implications for the development of GC LN metastasis.

### 3.9 High expression of RET in the NP group

**FIGURE 8:**
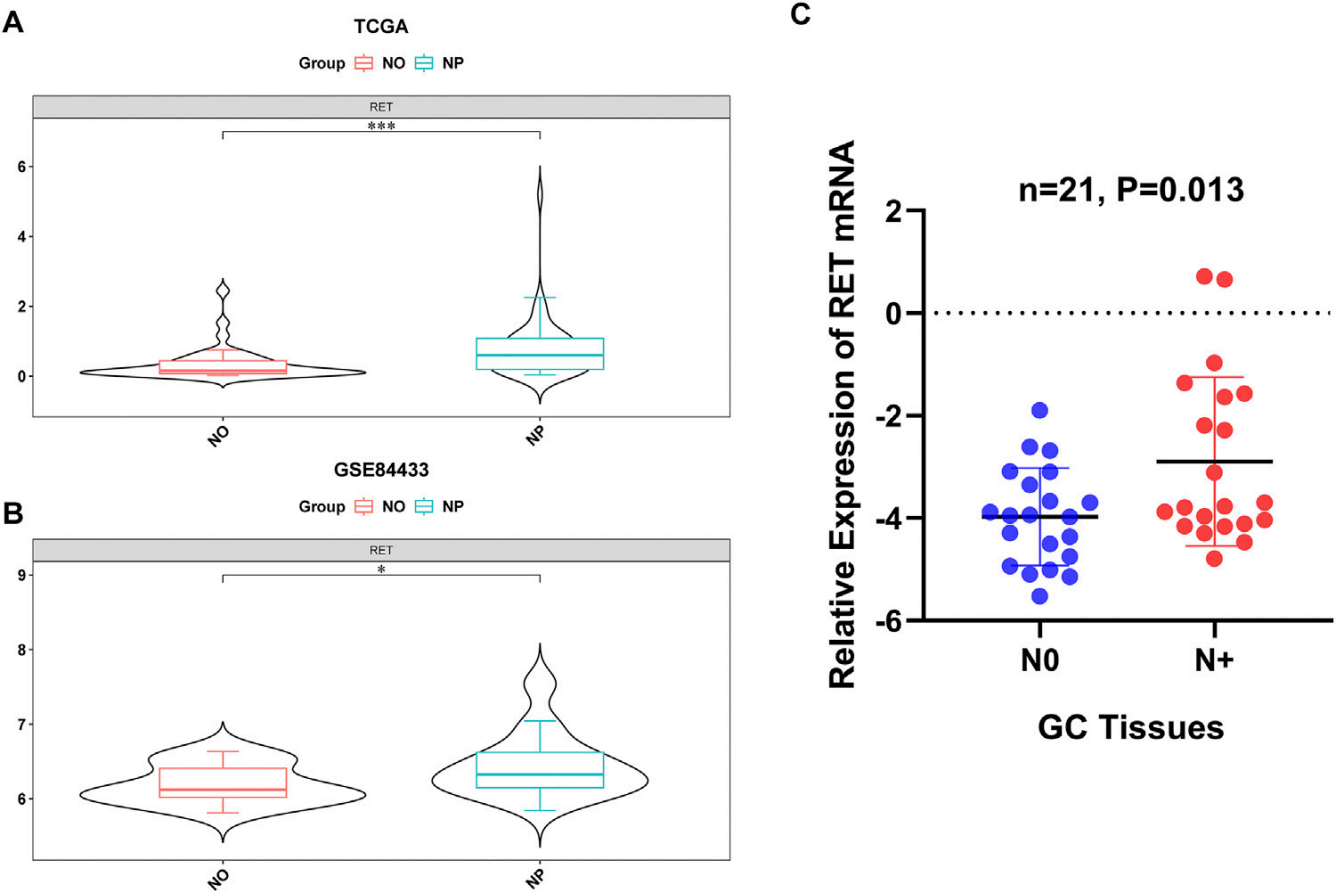
Validation of RET upregulation in LN metastasis. **(A,B)** RET mRNA overexpression in NP vs NO groups from the TCGA-GC-LN (*P* < 0.001) and GSE84433 (*P* < 0.05) datasets. **(C)** RT-qPCR confirmation in clinical specimens (*P* < 0.05).

In both the TCGA-GC-LN and GSE84433 datasets, RET was significantly upregulated in the NP group (Figures 8A,B). The RT-qPCR results of clinical samples also indicated a significant upregulation of RET in the NP group (Figure 8C), consistent with the bioinformatics analysis, further highlighting the importance of RET.

### 3.10 RET knockdown inhibits the progression in GC cells

**FIGURE 9:**
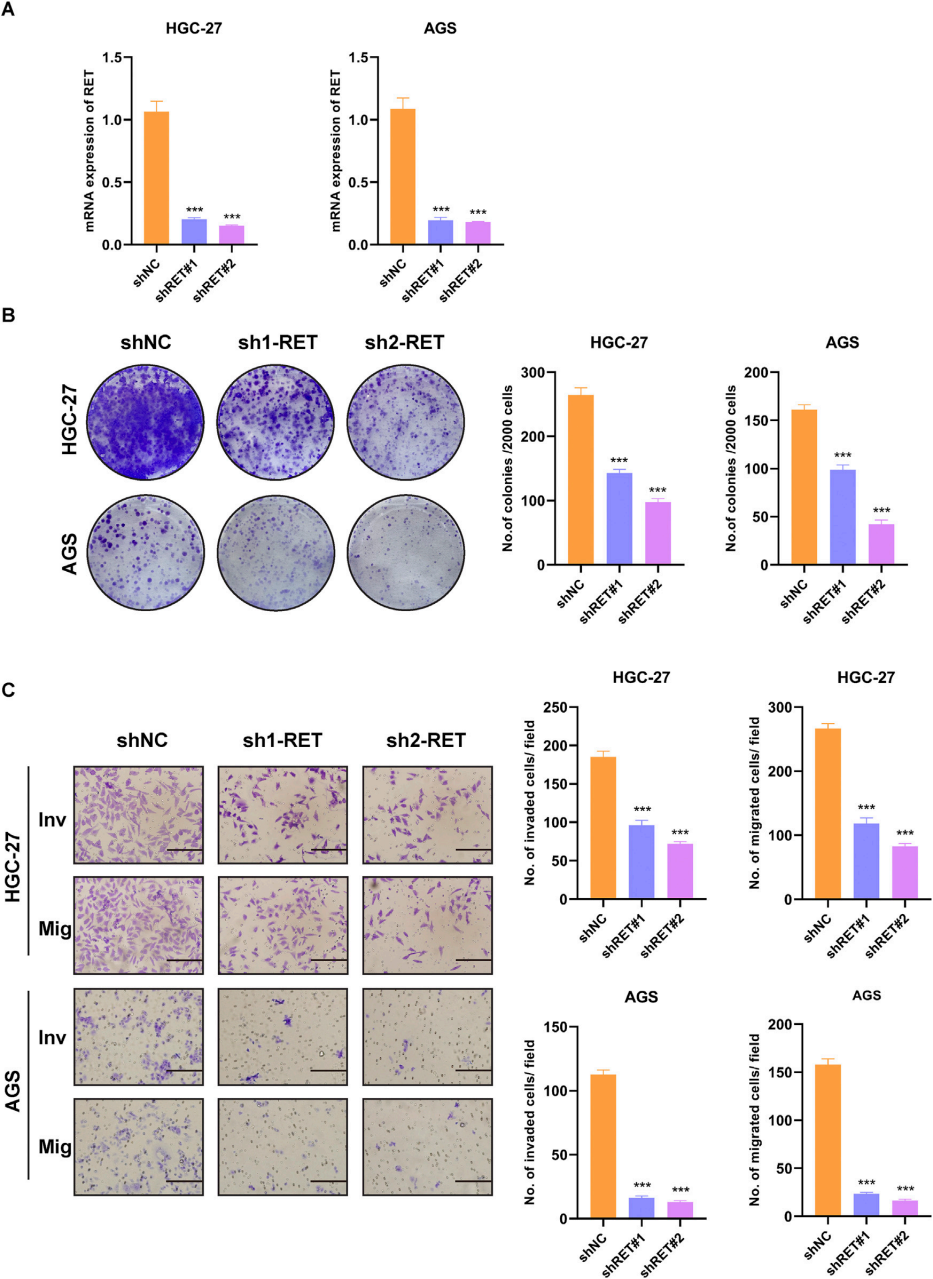
RET knockdown suppresses malignant phenotypes in GC cells. **(A)** RT-PCR validation of RET shRNA knockdown efficiency. **(B)** Colony formation assay. **(C)** Transwell migration and invasion assay (scale bar, 100 μm).

To examine the role of RET in GC cells, we employed shRNAs to deplete endogenous RET in HGC-27 and AGS cell lines. The efficiency of RET knockdown was validated through RT-PCR analysis (Figure 9A). RET-depleted GC cells exhibited a significantly reduced growth rate compared to control cells (Figure 9B). Furthermore, RET knockdown in GC cells markedly impaired cell migration and invasion (Figure 9C). Taken together, these findings indicate that RET plays a crucial role in promoting the progression of GC cells.

## 4 Discussion

The dysregulation of the calcium signaling pathway is critically implicated in tumor metastasis. Calcium ions (Ca^2+^) function as pivotal second messengers, influencing cell adhesion, motility, and the expression of key molecules via calcium-dependent proteins and calcium channels, thereby serving as a fundamental driving force for tumor metastasis. Aberrant calcium-dependent proteins result in reduced intercellular adhesion, facilitating the detachment of tumor cells from the primary site. In gastric cancer, diminished expression of E-cadherin is significantly correlated with lymph node metastasis, as its absence compromises cell-cell junctions and enhances the dissemination of cancer cells to the lymphatic system (Wu et al., 2005). Calcium channels, such as TRP channels, can activate signaling pathways including Wnt/β-catenin and PI3K/AKT through the Ca^2+^/calmodulin axis, promoting cytoskeletal reorganization and pseudopodia formation, thereby expediting directed cell migration (Liu et al., 2024). In this study, we acquired transcriptomic datasets of T1 and T2 lymph node metastatic gastric cancer from the TCGA and GEO databases. We identified calcium signaling pathway-related biomarkers, specifically RET, and offered novel insights into the treatment of T1 and T2 lymph node metastatic gastric cancer. This was achieved through comprehensive analyses, including enrichment analysis, immune microenvironment assessment, somatic mutation evaluation, drug sensitivity testing, single-cell analysis, and the construction of regulatory networks.

RET (ret proto-oncogene) proteins are integral members of the receptor tyrosine kinase family, predominantly situated on the cell surface (Ibanez, 2013). Upon binding of ligands, such as those from the glial cell line-derived neurotrophic factor (GDNF) family, RET proteins undergo dimerization and autophosphorylation, thereby initiating downstream signaling cascades, including the RAS-RAF-MEK-ERK and PI3K-AKT pathways (Ibanez, 2013; Veit et al., 2004; Zhong et al., 2023). These pathways are pivotal in regulating cellular processes such as growth, differentiation, proliferation, and survival. The interplay between RET genes and calcium signaling is manifested in three primary aspects: firstly, RET expression or activity is modulated by calcium signaling as a downstream effector gene (Gong et al., 2023); secondly, specific domains within RET proteins, such as the cysteine-rich domain (CRD), are involved in calcium binding, which is essential for their functional integrity (Bithia and DOSS C, 2025); thirdly, aberrations in RET, such as mutations, can disrupt calcium signaling pathways, consequently affecting cellular processes like differentiation, migration, and apoptosis, and potentially contributing to oncogenesis or neurodevelopmental disorders (Liu X. et al., 2023).

RET has been extensively investigated in the context of thyroid and breast cancers. RET variation is positively associated with lymph node metastasis in papillary thyroid carcinoma (PTC), and the anti-tumor immune response may contribute to lymph node metastasis induced by RET variation (Fussey et al., 2019; Huang Y. et al., 2024). Upon binding with its ligands, such as the glial cell line-derived neurotrophic factor (GDNF) family, RET activates downstream signaling pathways, including MAPK and PI3K, which facilitate tumor invasion and metastasis (Buchholz et al., 2021). Furthermore, RET contributes to the enhanced invasion and migration capabilities of cancer cells by modulating the epithelial-mesenchymal transition (EMT) process (Alqahtani et al., 2021; Zhao et al., 2020a). In the case of advanced gastric cancer, RET protein expression is significantly upregulated and is associated with increased tumor malignancy. Patients with gastric cancer exhibiting high RET expression tend to have a poor prognosis and reduced survival times (Buchholz et al., 2021). The involvement of RET in lymph node metastasis in T1/T2 stage gastric cancer has not been thoroughly elucidated in the existing literature. Our study presents novel findings regarding the potential role of RET in this context. Specifically, RET, identified as an oncogene, exhibits elevated expression levels in T1/T2 gastric cancer and demonstrates a significant correlation with lymph node metastasis and poor survival outcomes. These findings support the proposition of RET as a promising biomarker for early detection of gastric cancer metastasis and for guiding targeted therapeutic interventions.

Furthermore, our study identified significant variations in the infiltration levels of 14 immune cell types between the positive and negative cohorts of T1/T2 gastric cancer lymph node metastasis. Notably, the most pronounced differences in infiltration levels were observed in the Activated B cell, Mast cell, and Immature B cell groups. Subsequent analysis of the correlation between the biomarker RET and the immune cells exhibiting differential infiltration revealed that RET was most significantly positively correlated with Mast cells. Single-cell analysis indicates that RET is highly expressed in various cell types, including mast cells. Mast cells, as crucial effector cells within the innate immune system, display considerable heterogeneity within the tumor microenvironment and can influence cancer progression through mechanisms such as angiogenesis, regulation of cell migration, and immune modulation (Hanes et al., 2021). In gastric cancer, there is a marked increase in mast cell infiltration, which further escalates with tumor progression (Lv et al., 2018). The density of mast cells is correlated with angiogenesis, lymph node metastasis, and patient survival outcomes (Sammarco et al., 2019; Micu et al., 2016). Mechanistically, mast cells secrete angiogenic factors, such as VEGF-A, and lymphangiogenic factors, including VEGF-C and VEGF-F, which facilitate angiogenesis and lymphangiogenesis, thereby elevating the risk of lymph node metastasis (Sammarco et al., 2019). Additionally, through the activation of the SCF/c-Kit signaling pathway, mast cells enhance CCL-2 expression, which in turn stimulates the proliferation, migration, and invasion of gastric cancer cells while inhibiting apoptosis (Zhong et al., 2018). Furthermore, mast cells secrete TGF-β1, which modulates the accumulation of regulatory T (Treg) cells, thereby suppressing immune surveillance and promoting metastasis (Zhao et al., 2020b; Lv et al., 2024). Recent studies have demonstrated that the activation of mast cells through the IL-33/ST2 signaling pathway facilitates the aggregation of tumor-associated macrophages and enhances tumor angiogenesis and lymph node metastasis (Eissmann et al., 2019). These findings suggest that mast cells contribute to lymph node metastasis in gastric cancer by promoting mechanisms related to lymphangiogenesis and cancer cell activity. However, the specific role and mechanisms of mast cells in the early stages of tumor development remain inadequately understood. Our research indicates that mast cells are significant in predicting the risk of lymph node metastasis in T1/T2 stage gastric cancer. Further investigation is warranted to explore their potential as prognostic biomarkers.

Our study employed GSEA to identify 36 significant differential pathways between the NP group and the NO group, with particular emphasis on the activation of two oncogenic pathways: EMT and CSCs activity. The EMT pathway is critically involved in lymph node metastasis of gastric cancer, facilitating the transition of epithelial cells to a stromal phenotype and thereby enhancing their migratory and invasive capabilities. Clinical investigations have established a positive correlation between the expression of EMT markers and lymph node metastasis in gastric cancer (Pretzsch et al., 2022). This study further elucidates the pivotal role of the EMT pathway in lymph node metastasis during the T1/T2 stages of gastric cancer, thereby providing a theoretical framework for targeting the EMT process as a strategy to inhibit metastasis. Although the NP group did not show a significantly increased tumor mutation burden (TMB), the TP53 gene exhibited the highest mutation frequency, predominantly consisting of missense mutations. Mutations in TP53 can lead to impairments in the DNA damage response and genomic instability, thereby promoting cancer cell proliferation and the development of metastatic phenotypes (Tornesllo, 2025). Drug sensitivity analysis revealed that the NP group demonstrated reduced sensitivity to AKT inhibitor VIII and BIBW2992, suggesting that the AKT signaling pathway may contribute to the drug resistance mechanisms observed in patients with lymph node metastasis. In contrast, the NO group exhibited a poor response to NU.7441, which may be linked to alterations in its DNA repair pathway. These findings provide valuable insights for the development of personalized therapeutic strategies for patients with T1/T2 stage gastric cancer with lymph node metastasis.

This study utilized bioinformatics methods to identify the biomarker RET, which is associated with T1 and T2 lymph node metastasis in gastric cancer. It involved an analysis of the biological pathways related to the biomarker, its association with the immune microenvironment, molecular regulatory networks, and single-cell analysis. These findings offer novel insights that could inform the development of new therapeutic strategies for treating gastric cancer with T1 and T2 lymph node metastasis. However, the research is subject to certain limitations. It is based on bioinformatics analysis, and constraints pertaining to sample size and dataset may impact the generalizability of the findings. To enhance the reliability and clinical relevance of the research, future investigations should focus on increasing the sample size and conducting multicenter validation to ensure broader applicability of the results.

## Data Availability

The original contributions presented in the study are included in the article/Supplementary Material, further inquiries can be directed to the corresponding authors.
